# Aging as Adaptation

**DOI:** 10.1093/geront/gnad049

**Published:** 2023-04-25

**Authors:** Susan Watkins, Jayne Raisborough, Rachel Connor

**Affiliations:** School of Humanities and Social Sciences, Leeds Beckett University, Leeds, West Yorkshire, UK; School of Humanities and Social Sciences, Leeds Beckett University, Leeds, West Yorkshire, UK; Manchester, Greater Manchester, UK

**Keywords:** Collaboration, Cooperation, Feminism, Intergenerational, Theater

## Abstract

In traditional gerontological terms, adaptation is usually understood as the production of physical aids to mitigate the impairment effects caused by age-related disabilities, or as those alterations organizations need to make under the concept of reasonable adjustment to prevent age discrimination (in the UK, e.g., age has been a protected characteristic under the Equality Act since 2010). This article will be the first to examine aging in relation to theories of adaptation within cultural studies and the humanities. It is thus an interdisciplinary intervention within the field of cultural gerontology and cultural theories of adaptation. Adaptation studies in cultural studies and the humanities have moved away from fidelity criticism (the issue of how faithful an adaptation is to its original) toward thinking of adaptation as a creative, improvisational space. We ask if theories of adaptation as understood within cultural studies and the humanities can help us develop a more productive and creative way of conceptualizing the aging process, which reframes aging in terms of transformational and collaborative adaptation. Moreover, for women in particular, this process of adaptation involves engagement with ideas of women’s experience that encompass an adaptive, intergenerational understanding of feminism. Our article draws on interviews with the producer and scriptwriter of the Representage theater group’s play *My Turn Now*. The script for the play is adapted from a 1993 coauthored book written by a group of 6 women who were then in their 60s and 70s, who founded a networking group for older women.

In 1993, a group of six women in their 60s and 70s known as The Hen Co-Op, cowrote and published *Growing Old Disgracefully: New Ideas for Getting the Most out of Life.* A mixture of their life stories and advice, the book advised other women on how to live well as they aged, especially through creativity, friendship, and mutual support. Some 30 years later, two granddaughters of these original “hens,” a producer and scriptwriter in their 30s and 40s, respectively, adapted the book into a stage play, *My Turn Now*. Rather than a faithful adaptation of book to stage, *My Turn Now* was written collaboratively with women actors, as part of the Representage theater group’s project to “combat the invisibility and ageism experienced by women as they get older.” The devising process involved firstly reading the book, then an intensive week of improvising several scenarios, with the scriptwriter imaginatively reworking and consolidating the material into a more formal script. The result was a creative adaptation that drew together the book with the women actors’ personal and professional experiences of aging. More specifically, *My Turn Now* comprises a front-of-stage narrative, set in 1989–1991, inspired by the “hens,” and a “back-of-stage” narrative, set in the present-day, about the professional and personal lives of the four women actors playing the parts of the front-of-stage women. Each actor thus plays two roles each; for example, an actor may play a “Back Stage” TV soap opera star and a Front Stage retired teacher and union activist. Both parts are of similar ages and events on stage are split between the onstage performance and the “backstage” action, which takes place in a dressing room and occasionally the wings. This adaptation brought to life the changes and some enduring aspects of women’s aging across the generations: as such it offered a powerful and critical reflection on ageism and enabled intergenerational dialogue around the lived, specifically gendered, experiences of aging between the writers, actors, and their audiences.

This paper draws on interviews with the producer and scriptwriter to explore if the creative practice of adaptation can be used to create dynamic imaginations and new narratives of women’s aging. It has long been argued that these are needed if, as Segal argues, we are to “provide more nuanced thoughts on ageing” (2013, p. 18) than those provided by exhortations to remain youthful (denying age) or those that present age as a dreaded time of physical/cognitive decline, vulnerability, and social exclusion. The idea of active aging, the favored response to the challenges of an aging population, with its associated stress on maintaining independence, has clear limitations of its own. Endorsed by the World Health Organization, active aging refers to a suite of policies and initiatives aimed at reducing the impact and cost of predicted older populations, by encouraging and supporting people’s health, independence, and social inclusion for as long as possible (Boudiny, 2013). What emerges across active aging are “descriptive categories,” “positive visions,” and “prescriptive claims” that together craft a pervasive imaginary of future older citizens and the “good life they are expected to enjoy” (Pfaller & Schweda, 2019, p. 44). This framing neatly erases images of older age, the so-called “fourth age”—the “terminal place” for agency and choice-making (Gilleard & Higgs, 2011, p. 138); it denies the diversity and flux of the body’s capabilities as it ages (Calasanti et al., 2006); it contributes to the process of making actual aging bodies less visible and acceptable (Martin, 2012) and creates unrealistic expectations of aging and older life for young and older people alike. Segal calls for “richer versions of ageing” (2013, p. 19) that encompass cultural, bodily, and notably lived experiences of aging that scaffold our ability to stay “alive to life itself, whatever our age” (p. 4). Yet, the task of reimagining aging is confounded by internalized ageism, its prevalence in wider culture, and a widespread difficulty in imagining a future, older self (Jones, 2022). Creative approaches have been suggested as ways to help us think anew or askew at normalized ways of living (Jones, 2022; Richards et al., 2012). In this spirit, this article explores the possibility of reframing aging as a process of creative, improvisational, and collaborative adaptation.

## Rethinking Adaptation

The familiar layperson’s definition of adaptation is captured by the second of the *Oxford English Dictionary*’s seven different definitions of adaptation: “The action or process of adapting one thing to fit with another, esp. a new or changed environment.” Within gerontology, adaptation has usually been understood in “common-sense” or biological terms. Nikitin and Freund, for example, encompass both definitions:

The motivational approach focuses on the role of goals for successful aging. From this perspective, adaptation does not only encompass the adjustment of a person to changes in the availability of internal and external resources and demands of the environment, but also entails that persons proactively place themselves and shape their environment according to their goals. As such, similar to the usage of the term in biology, adaptation refers to a process of optimizing the fit between a person and the environment when faced with internal or external changes. (2019, p. 282)

A simple search for the term adaptation using the search facility on the landing page of *The Gerontologist* produced 3,462 results. Ordering by relevance (where the term appeared in the article title) demonstrated that adaptation was being understood predominantly in the same common-sense or biological terms. However, there were also a number of uses of the term as it is understood within occupational therapy, where, for example, “Occupational Adaptation (OA) is a theoretical framework used to facilitate people resolving real-world challenges through active problem-solving, using relative mastery as its measure” (McKay et al., 2021). Searching for the term adaptations (plural) produced articles focusing on technological advances and how these might be used by the geriatric population (e.g., Foster et al., 2016).

Within the arts and humanities, however, adaptation studies have now become an established field of study, with two international peer-reviewed journals. *Adaptation*, published by Oxford University Press, is linked to a professional association (the Association of Adaptation Studies) and organizes an annual conference, and the *Journal of Adaptation in Film and Performance* is published by Intellect Books. The websites for both journals give a sense of the richness and diversity of the field and also of how the term adaptation is understood. The *Journal of Adaptation in Film and Performance* focuses on “theater, film, and other media” and “the place of adaptation and translation within historical and contemporary cultures.” *Adaptation* includes interdisciplinary approaches from “Film and Screen Studies, Literary Criticism, Visual and Performing Arts, and New Media and Transmedia Storytelling. Topics range from familiar examples of adaptation such as novel and stage to screen adaptations to less recognised forms like remakes, fan fiction, and themed experiences. The journal encourages research that provides global perspectives on adaptation.”

One of the important early debates in the field was about fidelity (to the source text). Recently, there has been a definite move away from fidelity criticism (the issue of how faithful an adaptation is to its original) toward thinking of adaptation as a creative, improvisational space. As Linda Hutcheon puts it:

When we call a work an adaptation, we openly announce its overt relationship to another work or works […] An adaptation’s double nature does not mean, however, that proximity or fidelity to the adapted text should be the criterion of judgement or the focus of analysis. For a long time, fidelity criticism, as it came to be known, was the critical orthodoxy in adaptation studies […] Today that dominance has been challenged from a variety of perspectives […] and with a variety of results. (2013, pp. 6–7)

Hutcheon calls the discourse of fidelity “morally loaded” and “based on the implied assumption that adapters aim simply to reproduce the adapted text” (p. 7); in fact, adaptation may include the “urge to consume and erase the memory of the adapted text or to call it into question” rather than to “pay tribute by copying” (p. 7). In practice, adaptations exist in relationship to the adapted text; therefore, they are inevitably double. However, questions of fidelity are not the only questions that can be asked about adaptations. If we move beyond issues of fidelity, then what should we consider in their place? Hutcheon argues that “the act of adaptation always involves both (re-)interpretation and then (re-)creation” (p. 8); this is sometimes referred to as “reappropriation.”

Julie Sanders points out the need to view the process of adaptation as collaborative: “adaptation and appropriation, as both procedure and process, are celebratory of the cooperative and collaborative model of creativity” (2016, p. 15). Like Hutcheon, she argues that concepts of in/fidelity are unhelpful, claiming instead that we need to move “towards more malleable and productive concepts of creativity” (p. 18). Rather than endlessly alluding to classical, canonical source texts in a respectful process, “adaptation can be oppositional, even subversive. There are as many opportunities for divergence as adherence, for assault as well as homage” (p. 19). One of the problems with common-sense assumptions about the relationship between an original text and its adaptation is the dominance of a language of accuracy, and the primacy of the source text, so that “the discussion will therefore always be couched in terms of difference, lack or loss” (p. 22). In fact, it is impossible to assess the extent or otherwise of fidelity, both because of changes in medium (in the case of novel to film, for example) but also because the original text is multiple, shifting, and complex. As Sanders has it: “many of the so-called ‘original’ texts we are handling […] are highly labile, adaptive patchworks themselves” (p. 27).

However, Geraghty argues that the issue of fidelity cannot be dismissed, because it “matters to the viewer” (Geraghty, 2008, p. 3). In her 2015 defense of fidelity, Hermansson acknowledges this point, and claims that fidelity criticism should be understood as “one essential tool in the intertextual toolbox of adaptation studies” (2015, p. 147). She argues for the importance of “opening out the study of the fidelity relationship simultaneously with consideration of other intertexts in the web (or layers in the palimpsest)” (pp. 156– 157). Adaptation studies, then, now focus on adaptation as a creative, collaborative process in which improvisational, even irreverent appropriation of the source text might take place, but in which the relationship with the source text is still worthy of discussion.

Theater offers precisely such a space or process for creative appropriation. Although in the case of scripted theater, the script provides a set of constraints that each performance follows, there is considerable scope for variance, or adaptation, from performance to performance. Sanders argues that “performance is itself an inherently adaptive art; we might even argue that each individual performance is an adaptation […] drama embodies within its own conventions an invitation to reinvention” (p. 60). Connor has written previously about her own experience, as a practitioner, of adapting, devising, and coproducing Strindberg’s *A Dream Play* (1901) for a contemporary audience in a café in the center of Manchester. She concluded that there is freedom in innovation and in the “transformative spaces that allow for multiple authorship, for creativity and play” (2018, p. 81).

This creative freedom can help create alternative accounts of aging in theater. Previously published work on theater in *The Gerontologist* focuses on older people’s theater-going and its value to well-being (Meeks et al., 2018) and theater arts initiatives in residential care (Pauluth-Penner, 2016). There is less work on older people’s participation in theater *making* (one important exception is the work of Lipscomb (2015, 2016a)). However, theater making allows for the generation of new conceptions of aging.

Lipscomb and Marshall introduce the concept of age “*as a performative*” (emphasis original; 2020, p. 1), arguing that “each of us performs the actions associated with a chronological age minute by minute, and that the repetition of these performances creates a so-called reality of age both for the subject and for those who interact with the subject” (p. 2). Lipscomb (2016b) also discusses the prominence of intergenerational relations and understanding in senior theater groups and their productions (pp. 179–180). Mangan’s (2013) analysis of the Western dramatic canon and contemporary British theater unearths how plays and performances are capable of both reinforcing and resisting familiar age ideologies, particularly the decline narrative. Davis Basting (1998) focuses on how eight different theatrical performances from the mid-west of the United States value old age as an important stage of life “that links generations, that is engaged in both the present and the past, and that is constantly changing” (p. 2). There have also been attempts to develop new, positive representations of aging by enhancing the visibility of older women on stage (Casado-Gual & Schevchenko-Hotsuliak, 2021), and exciting interpretations that foreground stronger pro-aging narratives while disrupting conventional chronologies of time and aging (see Henderson’s (2016) account of a Vancouver production of *King Lear*).

It is, however, the potential for the creativity and innovation *between* actors, audiences, and writers that can encourage awareness of and resistance to internalized ageist beliefs (Black & Lipscomb, 2017) to “expand the discourse about ageing” (Gatt, 2023). Casado-Gual (2021), for example, examines theater companies that work to connect local communities with artistic and academic institutions as sites where different meanings of age can be circulated and integrated. Casado-Gual concludes that such companies produce an affirmative discourse of aging that questions conventional binary (decline/success) narratives of aging. Similarly, Gillespie (2019) examines renowned company Split Britches’ production of *Unexploded Ordnances*. This production included audience interaction by the oldest members of the audience to create an interactive discussion about current crises to encourage thinking beyond finite endings. In this paper, we offer some insight into the practices of theatrical adaptation to help open up our critical and creative grasp of how alternative accounts of aging can be produced.

## Method

In 2019, two interviews were conducted with the producer and scriptwriter of the play *My Turn Now*. The research project was approved by the University’s Ethics Committee. The interviews were unstructured, although they started with a prompt asking the interviewees why they were involved with the play and their creative process in creating the play. The interviews were transcribed and analyzed by drawing upon Braun and Clarke’s (2006) step framework for thematic analysis. Familiarity with the data is a key aspect of Braun and Clarke’s approach to thematic analysis; accordingly, the transcript was read through several times while listening to the audio recording. We then worked through the transcript to produce initial codes. We used a manual method of highlighting the transcript, firstly independently so we could check coding reliability, and then as a team. The codes were herded into themes, which were reviewed and redefined in turn. For the purposes of this paper, we focus on an overarching theme we have labeled “aging as adaptation,” which incorporates subthemes of (i) adaptation and iteration, (ii) adaptation and collaboration, and (iii) adaptation and intergenerational feminism, which we take in turn below.

(i) Adaptation and iteration—fidelity versus creativity

Perhaps the most interesting and innovative aspect of *My Turn Now* is the interaction between the front-of-stage and “backstage” characters in relation to the book. Their bleeding into one another generates a metatheatrical quality that makes the connections and comparisons between previous and current attitudes to gender and aging very clear. This innovation was necessitated by the book itself: its own unconventional form (“there isn’t really a narrative in the book”) meant that they “decided very early this is going to be an ‘inspired by’ rather than an ‘adaption of’ or a ‘based on’ even” (Producer). However, the main inspiration for the front/backstage arose from group readings and discussions of the book:

We decided to have the reading with a group of actresses over sixty above a pub in south London and for that we got them to read sections of the original book and then to discuss what they were hearing. And I think what came up in their discussions was amazing. As a group it was full of insight and funny and poignant and I was like: oh my God there is so much here! There is so much gold in this conversation between the original text and the lives of these women today, and how can we use that dramatically? So that’s when I came up with the idea of the front stage backstage thing. (Scriptwriter)

The “gold” refers to the opportunities for dialogue with the book and across several professional and personal experiences of age and aging as contemporary women related experiences from the past with their own. According to the producer, the work of improvising and then devising allowed the actors to incorporate

how they think their careers have compared to their male counterparts, how they think their casting has changed as they’ve got older, any experiences of sexism or ageism that they’ve experienced in the industry. And they were amazingly open about that and really shared a lot of very personal, sometimes quite painful, stuff.

We have written elsewhere about the adverse impact of ageism on the careers of women actors, as gendered age stereotyping produces a striking reduction in the quantity and complexity of roles for women actors as they enter their 40s (Raisborough et al., 2021). The intersections of sexism and ageism may therefore be particularly pronounced for women actors, many of whom are forced out of the profession as roles dry up: with that loss goes the ability to earn a living (Nobis, 2015). The “personal” “painful stuff” suggests how much these connected forms of oppression become embodied and affectual for older women, particularly those who make a living from acting/embodying a different person. Clarke and Korotchenko, for example, argue that there is some evidence to support the idea that “the means by which older women evaluate their bodies shifts from appearance to physical function” (2011, p. 497). As women actors age, painful experiences of sexism and ageism are increasingly lived “on the body.”

Significantly for our purposes, the choice not to pursue a faithful adaptation, while allowing creative “gold” did nonetheless introduce two issues. The first was the play’s relationship to the original book and the second was the skill set required to create new narratives. In terms of the first, the producer explained that

[…] So then it becomes more of a conversation about what’s changed, what hasn’t changed. And I was excited by it dramatically. […] So then it was hard because I know all of these women and I feel all this responsibility and fidelity to the women.

Concepts of responsibility and fidelity here speak to the producer’s concern to air the various voices of the wider project: a creative tension developed from the need to represent the hens and to air the experiences of the contemporary women actors. As we discussed above, the nature of the original book (collaboratively written and with no clear narrative) combined with the dynamic discussions and improvising sessions during the first reading to open up new creative opportunities. The reworkings of the script through discussions and improvising possible scenarios for the stage enabled curious recharacterizations that blended the hens’ voices with those of the actors to create what the scriptwriter called “composite characters”:

I have to create composite characters and different characters and I can take loads of elements of the original women, but I can’t write this if it’s them, because I just can’t do what I need to do as a writer. So that was another kind of massive relief. Then I started to create these fictional characters and I think around that time, because it had been so generative and rich working with these actresses, I was like: ‘well let’s do that more’, let’s build a process where we are working in a semi-devised way.

It was through the development of composite characters (characters composed of more than one real life or fictional individual) and the process of devising (collaborative creation by the performers of a new piece of theater incorporating their life experience without a preexisting script) that once marginalized accounts of aging could be aired. Both interviewees were aware of the mainly middle-class, heterosexual Whiteness of the original hens. Both reflected on the lack of diversity in contemporary theater for women of color, sexuality, and class as they intersected with aging. It was this that motivated their political desire to create theater that rendered visible otherwise invisibilized power relations in order to generate transformation. The producer expressed this as a responsibility:

I think we’ve got a responsibility to make plays about diverse people for more diverse audiences. So this gives us the option of casting actresses from different ethnicities and then discussing that in the play, as well as talking about it and talking about the issues of representation in so many different ways in this industry, and kind of putting it all out in the open.

Here we see that freedom from fidelity *allows* the process of adaptation to become transformative. It is precisely this point that Sanders makes in her discussion of adaptations of Shakespeare, where “the transformation involved in seeing things from a different point of view is a driving force” (p. 61). The driving force of the adaptation process pulls on the reflexive positionings of the producer and scriptwriter, who are drawn to think critically about their ability to create diverse composite characters from their situated positions of race and age privilege. As a younger, White woman, the scriptwriter spoke of the need to create more diverse representations yet of the “ethical questions” this posed for her:

I guess there was a lot for me as a white woman, writing a woman of colour, and I think that’s raised, and still has raised that I haven’t resolved, lots of ethical questions in me. And I decided I wanted to represent a woman of status, so the woman of colour character to be someone with status. And then I started to look at well who, just looking, googling, so who the women of colour in the 1980s who would have been sixty or seventy. And there were very few. Obviously there were very few prominent women of colour at that point in history.

The scriptwriter here articulates a clear sense of the ways in which the processes of adaptation and collaboration drew on the different experience of the woman actor of color. Despite concerns about the ethics of a White person writing a person of color’s story, the scriptwriter’s doubts about this were overtaken by feeling a clear responsibility to include different and diverse stories and to include an awareness of the intersectional nature of forms of dis/privilege. Researching the stories of prominent women of color in the 1980s allowed the scriptwriter to offer a more nuanced understanding of this: the character is disprivileged in terms of race, but not in social status. Yet the decision to understand the play as “inspired” by the book allowed a stronger collaborative and affective bond between actors, producer, and scriptwriter as part of a shared creative encounter. And too a stronger political motivation to confront power relations in the cultural industries. The devising work of the actors, which drew on their own experiences of sexism and ageism in the theater and other cultural industries, was centered.

(ii) Adaptation and collaboration

It is clear that the collaborative creation of *Growing Old Disgracefully* means that it was already, in Sanders’s terms, a “highly labile, or patchwork” text (p. 27) composed by multiple authors working together. The construction of the script for *My Turn Now* echoed that process, as it involved (as we have seen) the devising work of the actors, plus the work of the scriptwriter and producer. At one point, the team even returned to the original hens

Mainly for the actresses to ask them questions, ask anything that they weren’t sure about that had come up in the process for them about the process of writing the book and or them meeting and writing the book and then what happened afterwards. (Producer)

This return was not, however, about authenticating the script in relation to the original source text. Although the three original hens have seen the play in performance, they were not asked for their feedback, as the producer felt that “it’s not that kind of relationship I don’t think that we want.” Collaborative working was not, then, a process of respectful homage offered by the younger women to their grandmothers’ original text but instead offered the opportunity for creative, improvisational, even disruptive work to take place in creating an adapted text.

The scriptwriter talked about the pleasures of collaborative work, commenting that “I also don’t love the isolation of the writing process and the huge pressure it puts on you as an individual, so I was really up for playing and doing something collaborative.” The idea of playing with writing and the adaptation process thus becomes a positive and rewarding alternative to individual authorship. Moreover, the lessons of collaborative work and understanding of adaptation as a collective rather than individual process were also connected to interviewees’ understanding and awareness of aging. The scriptwriter mentioned the “life course approach to thinking about aging. So instead of thinking about discrimination as you get old and old being a kind of ‘post-X’, thinking about actually how do we think about the expectations and prejudices we have about our age at any point in life.” Here, aging is something that everyone experiences, a part of everyone’s imaginary and actual narrative of self, rather than a point that an individual reaches “post” the age of “X.” This rethinks age as something that affects everyone collectively. Here the scriptwriter suggests a much more flexible, culturally and socially inflected understanding of age-based identity within the life course.

The life course approach to aging has been defined as a “negotiated, unstable assemblage of ideas and perceptions in which ‘age’ competes with other imperatives such as gender, class and ethnicity” (Hockey & James, 2003, p. 4) and in which, as Hockey and James argue, the key question is “how do we come to know that we are ageing?” (p. 3). For the scriptwriter, the aim of the project, then, was to create a collaborative, plural vision of the process of “coming to know that we are ageing”: “The ambition of this kind of project isn’t to replace that with another singular vision of what it is to be an aging woman, but to show the multiplicity of different ways. I mean there is an infinite number of different ways that we will age but to show more of that.” The scriptwriter here alludes to the need in the wider culture for an “infinity” of visions of aging. These are necessary not only to counter the damaging singular narratives that currently exist, but also to create collective engagement in and responsibility for those narratives. The collaborative adaptation process experienced by both scriptwriter and producer with the women actors and original hens generated an awareness of this need and how it might be met by sharing collective approaches to the process of aging among those of different generations.

(iii) Intergenerational feminism as adaptation

The process of adapting their grandmothers’ book allowed both scriptwriter and producer to learn about the position of women decades earlier and compare it with their own:

most of them had got married very young, had children very young and then in their forties and fifties got divorced, or a couple of them their husbands passed away and they suddenly, in this kind of what they thought was going to be their last chapter in life, suddenly had more freedom than they’d ever had before. And were going travelling and having all these adventures that they never dreamed of and this whole kind of second life.

A “second life” of “freedoms” for their grandmothers followed a confined and restricted early experience. This transformation was initially understood in relatively straightforward terms, perceiving those life alterations wrought by the aging process as the agent of change. The imaginative comparison between mother, daughter, and (implicitly) granddaughter focuses on the new opportunities for the mothers’ generation in comparison to the grandmothers’. The producer remarked: “The difference between the generation before them and the generation after them is just enormous. And these women all would have seen […] their daughters having freedoms that they couldn’t have imagined when they were teenagers or in their twenties or thirties.”

In 1999, Woodward wrote of the need for “generational consciousness of older women” (p. 163). Feminist scholarship and activism had been open to the criticism that aging was not an issue until the feminists of the 1960s and 1970s themselves grew old (Brennan, 2005; Rubenstein, 2001), even if it has since begun to address the topic in a substantial critical literature. Rather than wait for younger women to grow older and age into an awareness of the particular intersections of gender with age, the experience of adaptation involved in creating *My Turn Now* offers hope for what Purvis refers to as the “challenges” of intergenerational feminism (2004), in which a more critical stance on normalized power relations is created, along with the capacity to disrupt those relations with new accounts and vision of aging (Jones, 2022), It is important then, to explore how feminism can act as such as instigator of change if we think in intergenerational terms. Both interviewees demonstrated an understanding of this. The producer commented:

And I think what’s really interesting, and the reason that we decided to call the play *My Turn Now*, is this thing that happened for that generation, where they had grown up in such a different world to the one that we know now.

Seeing the hens’ growing awareness of their daughters’ increasing freedoms and the different world that they were able to enjoy was clearly a key impetus behind the play and its title. However, the relationship between the generations of women involved in this project was also important. The scriptwriter commented on her

efforts to understand where I fit in to this story. How do I place myself? Just in terms of how can this be a multi-generational story? I’m in my early forties. […] There’s a range of ages of women involved in the production of this show.

There is a clear awareness of the relationship between her own position in comparison with that of her mother and grandmother. This also extends into reflections on feminism and its own historiography. Growing up in the late 1980s and 1990s, she remarked: “That’s not a bit of the history of feminism that gets talked about very much. That time period—the 1980s and 1990s—because in some ways as a young woman in that time it was quite a regressive phase, in that it was laddishness.” In relation to her awareness of feminism at that time, the scriptwriter was “brought up being told we had it all, you know, we’ve had first-wave second-wave feminism, so it’s [the late 1980s/early 1990s] this funny bit in the history of feminism.”

Growing up without a lived experience of feminism that felt real and directly applicable during the “funny bit” of the late 1980s/early 1990s was clearly a loss, but one that was only realized, confronted, and worked through later on, during the adaptive process of working on *My Turn Now*. The play itself and the process of creating it “help[ed] to make the different histories of feminism more visible.” She continued:

All those women’s consciousness-raising groups were something that I only knew about through Grandma, *but it’s never been reflected back*. I know a lot of us who grew up and came of age in the 1980s and 1990s. I feel pretty angry about the kinds of messages I got about being a woman and so it’s nice to show what else was happening during that time [our emphasis]

The words “reflected back” here are richly suggestive. The play can “reflect” on the period of the 1980s and 1990s and the present moment for the original “hens,” for those developing the play and for the audience. The reflective relationship works in more than one direction. In fact, the moment of the Hen Co-Op, *Growing Old Disgracefully* and the moment of the present-day do more than mirror one another: they exist in conversational dialogue, in an adaptive relationship that is about multiple different resonances and consonances between women’s understanding of feminism/s and also about women’s intergenerational experiences of aging.

## Discussion

It is becoming axiomatic within age studies that current narratives about aging are damaging and restrictive and fail to generate the flexibility and creativity that is required of us all as we age. Even a feminist aging project explicitly focused on transformative visions of aging found it hard to create new visions of old age “if they did not connect to these widely-known existing ideas about what constitutes a good old age” (Jones, 2022). Jones continues however to argue that “research and other creative projects can endeavour to extend the range of what is imaginable” (no page). The process the Representage theater group went through when adapting *Growing Old Disgracefully* to create *My Turn Now* is an example of just such a project: it produces a transformative narrative for aging, but one that works to “reappropriate” (in Sanders’s terms) a source text, rather than seeking to create entirely new visions that are not rooted in a lived history.

There are many benefits to moving beyond fidelity to the source scripts of aging, even if those sources do still, as Geraghty suggests, “matter.” Rather than being constrained by previous fixed ideas about the aging process, it becomes possible to improvise, in a more playful way, new aging identities that are not solely based on what Sanders, in the context of adaptation studies, refers to as “difference, lack or loss” (p. 22). Understanding aging in terms of adaptation also stresses the importance of *collaborative* creation, rather than individual authorship. Sanders claims that “adaptation and appropriation, as both procedure and process, are celebratory of the cooperative and collaborative model of creativity” (2016, p. 15). The play *My Turn Now* exists in the form it does because of the collaboration of the scriptwriter and producer with the original hens, as well as with the actors who took on the roles and brought their own stories into the mix during the devising process. This collaborative understanding of the artistic process can, we suggest, be extended out to our understanding of the aging self, or subject. As we age, we draw on the experiences and stories of those around us. This is a continuous, iterative, negotiated, and transformational process; it moves beyond any necessity to preserve absolute fidelity to our youthful selves, while it acknowledges that previous versions of the self “matter.” It can offer a nuanced, lived practice of rewriting and change that is able to accept contingency.

Understanding aging in terms of adaptation also offers a positive alternative to the stress on maintaining independence as we age, meaning that *dependence* is then no longer viewed negatively. Instead, dependence becomes germane to the formation of any lived identity in the world. Segal argues, for example, that dependence is key to the construction of all identities, rather than solely to aged identity. She admits that fears about dependency are central, in her experience, to ideas about old age, but makes the claim that “differing modes of dependence are essential to the human condition” (p. 35). The scriptwriter of *My Turn Now* talked about the pressure the isolation of the usual solitary writing process placed on her as an individual. A similar pressure can be seen in the idea of maintaining independence as we age. Independence is the implicit necessity behind ideas about “healthy” aging; moreover, independence individualizes the aging process, with blame and culpability attached to the idea of dependence. The scriptwriter found a positive alternative to the usual writing practice in collaborative writing, which she termed “playing.” Understanding adaptation as a playful, collaborative artistic practice allows us to reconceive of aging in the same way: as a shared process that we are all continually experiencing, a process that should highlight the positive ethical aspects of dependence on others.

Reconceiving of aging as a process or practice of adaptation also recognizes the importance of intergenerational experience and promotes an intergenerational understanding of feminism for women as they age. The scriptwriter talked about “knowing through Grandma,” but also about not hearing about her own experience in official feminist narratives. She wanted to make “more visible” the “different histories of feminism” and felt that the play achieved that, particularly in the opportunity that the adaptation process offered to include the experiences of women of color and contest the White, middle-class voices of the original hens. The scriptwriter felt that the aging process demands a reengagement with feminism: on reaching her early 40s she wanted to understand where she fitted in and how to place herself, but also her grandmother and mother, within the “story” of feminism. It was particularly important for her to be able to reflect on and position her experience growing up in the late 1980s and early 1990s, what she referred to as the “funny bit” in the history of feminism. Purvis claims that generational differences can function to “proliferate animosity and divisiveness among feminists” and obscure what are actually political, rather than age-based differences (p. 106). She argues for the need to move beyond linear chronology. Many older women writing about aging have written about the new apprehensions of time that growing older brings. Lively, for example, reflects on the “new and disturbing relationship with time” that aging provides (2013, p. 43). The work of adapting *Growing Old Disgracefully* into *My Turn Now* meant that the women involved reached an awareness of the intersections of gender, age, and other power structures earlier than they otherwise might have done. It also meant that they saw through simple narratives about feminism that rely on linear chronology, whether that be in terms of waves or generations.

## Conclusion

Our analysis of interviews with the scriptwriter and producer of *My Turn Now* makes clear the importance of reconceptualizing narratives of aging in terms of contemporary cultural theories of adaptation. Adaptation has the potential to provide a much-needed new resource, given that aging is usually viewed in terms of decline or “healthy aging,” narratives that both privilege independence and deny and “other” infirmity and dependence. Adaptation as conventionally understood within gerontology reinforces those ideas, as our earlier discussion made clear. Drawing on theories of adaptation as understood within the arts and humanities and cultural studies allows us to begin to rethink experiences of aging. Adaptation in these disciplines stresses collaborative creation (of art) rather than individual authorship. This collaborative understanding of the artistic process can also be extended out to understand the aging self, or subject. A collaborative, cooperative construction of the aging self also offers a positive alternative to a focus on independence, Our case study shows that the practice of adaptation has the potential to reframe dependence as germane to the ethical formation of a lived identity in the world. Intergenerational experience becomes part of such a collaborative construction of self and for women it is an emerging way of engaging with the different “stories” provided by feminism. Looking forward and back across different generations of women allows consideration of both continuity as well as issues of difference (particularly in relation to race and sexuality, for example) to be addressed more openly. Understanding aging in terms of adaptation allows for a lived practice of negotiation, collaboration, creativity, dialogue, and contestation for the aging subject.

The producer offered a craft metaphor to sum all this up:

She [the producer’s grandmother] was very into crafting as well, doing knitting and sewing and tapestry and everything. And she talks about how in that kind of thing the threads all kind of knit together, and her hands feeling the thread and looking at the colour and how they all intertwine, and each thread is its own thread in its own right and it’s its own colour and it doesn’t matter how much you twist it up with a different thread it is still its own thread, but when you’ve got them all threaded together and then you step back and it’s this beautiful picture that all these unique threads have come together to make. And that’s how she talks about finding these women in later life and having this really fulfilling uplifting friendships for the first time, where she felt like she was her own person, but she had this network of strength around her that she was getting from other women, which maybe she hadn’t had in her romantic relationship.

The craft metaphor is a familiar one within feminist theory and practice (Harrison & Ogden, 2020); like a tapestry, an adaptation consists of many elements in conversation. Here, we offer it as an alternative to more familiar ideas about aging that have bedeviled the lived experience of getting older.

## Data Availability

The authors do not report data and therefore the preregistration and data availability requirements are not applicable.

## References

[CIT0001] Basting, A. D. (1998). The stages of age: Performing age in contemporary American culture. University of Michigan Press. doi:10.3998/mpub.15441

[CIT0002] Black, K., & Barnes Lipscomb, V. (2017). The promise of documentary theatre to counter ageism in age-friendly communities. Journal of Aging Studies, 42, 32–37. doi:10.1016/j.jaging.2017.06.001 28918819

[CIT0003] Boudiny, K. (2013). Active ageing: From empty rhetoric to efficient policy tool. Ageing and Society, 6, 1077–1098. doi:10.1017/S0144686X1200030X PMC3728916 23913994

[CIT0004] Braun, V., & Clarke, V. (2006). Using thematic analysis in psychology. Qualitative Research in Psychology, 3(2), 77–101. doi:10.1191/1478088706qp063oa

[CIT0005] Brennan, Z. (2005). The older woman in recent fiction. McFarland.

[CIT0006] Calasanti, T. M., Slevin, K. F., & King, N. (2006). Ageism and feminism: From “et cetera” to center. NWSA Journal, 18(1), 13–30. doi:10.2979/NWS.2006.18.1.13

[CIT0007] Casado-Gual, N. (2021). Exploring old age through the theatre: Three British senior theatre companies. In A. Hülsen-Esch (Ed.), Cultural perspectives on ageing (pp. 99–112). De Gruyter. doi:10.1515/9783110683042-007

[CIT0008] Casado-Gual, N., & Schevchenko-Hotsuliak, I. (2021). Disrupting temporalities: Multiplying the self: An age-studies approach to two contemporary plays. Alicante Journal of English Studies/Revista Alicantina de Estudios Ingleses, 35, 75–97. doi:10.14198/raei.2021.35.04

[CIT0009] Clarke, L. H., & Korotchenko, A. (2011). Aging and the body: A review. Canadian Journal of Aging/La Revue Canadienne du vieillissement, 30(3), 495–510. doi:10.1017/S0714980811000274 PMC4072651 24976674

[CIT0010] Connor, R. (2018). Retranslating Strindberg: Adaptation, (re)location and site-related performance. Journal of Adaptation in Film and Performance, 11(1), 71–83. doi:10.1386/jafp.11.1.71_1

[CIT0011] Foster, S., Schrader, A., Wei, J. Y., & Azhar, G. (2016). Adaptations in the age of technology in seniors. Gerontologist, 56(3), 528. doi:10.1093/geront/gnw162.2135

[CIT0012] Gatt, I. (2023). Combatting ageism through participation in a theatre-making process and performance. In A. Kárpáti (Ed.), Arts-based interventions and social change in Europe. Routledge Advances in Art and Visual Studies. Routledge.

[CIT0013] Geraghty, C. (2008). Now a Major Motion Picture: Film adaptations of literature and drama. Rowman.

[CIT0014] Gilleard, C., & Higgs, P. (2011). Frailty, disability and old age: A re-appraisal. Health, 15(5), 475–490. doi:10.1177/1363459310383595 21169203

[CIT0015] Gillespie, B. (2019). Detonating desire: Mining the potential of ageing in Split Britches’ *Unexploded Ordnances (UXO)*. Performance Research, 24(3), 89–98. doi:10.1080/13528165.2019.1583985

[CIT0016] Harrison, K., & Ogden, C. (2020). ‘Knit “n” natter’: A feminist methodological assessment of using creative ‘women’s work’ in focus groups. Qualitative Research, 21(5), 633–649. doi:10.1177/1468794120945133

[CIT0017] Hen Co-Op, The (1993). Growing old disgracefully: New ideas for getting the most out of life. Piatkus. 10.7748/eldc.5.6.12.s11 8298592

[CIT0018] Henderson, J. (2016). Challenging age binaries by viewing *King Lear* in temporal depth. Theatre Research in Canada, 37, 42–61. doi:10.3138/tric.37.1.42

[CIT0019] Hermansson, C. (2015). Flogging fidelity: In defense of the (un)dead horse. Adaptation, 8(2), 147–160. doi:10.1093/adaptation/apv014

[CIT0020] Hockey, J. & James, A. (2003). Social identities across the life course. Palgrave Macmillan. doi:10.1007/978-1-4039-1399-9

[CIT0021] Hutcheon, L. with O’Flynn, S. (2006; 2013), A theory of adaptation (2nd ed.). Routledge. doi:10.4324/9780203957721

[CIT0022] Jones, R. L. (2022). Imagining feminist old age: Moving beyond ‘successful’ ageing? Journal of Aging Studies, 63, 100950. doi:10.1016/j.jaging.2021.100950 36462912

[CIT0023] Lipscomb, V. B. (2015). Performance theory for senior theatre: Agelessness on stage. Gerontologist, 55(2), 195. doi:10.1093/geront/gnv553.06

[CIT0024] Lipscomb, V. B. (2016a). Documentary playwriting: Reflecting/revising community attitudes toward ageing. Gerontologist, 56(3), 256. doi:10.1093/geront/gnw162.102724793646

[CIT0025] Lipscomb, V. B. (2016b). Performing age in modern drama. Palgrave Macmillan.

[CIT0026] Lipscomb, V. B., & Marshall, L. (Eds.). (2020). Staging age: The performance of age in theatre, dance and film. Palgrave Macmillan.

[CIT0027] Lively, P. (2013). Ammonites and leaping fish: A life in time. Penguin.

[CIT0028] Mangan, M. (2013). Staging ageing: Theatre, performance and the narrative of decline. Intellect. doi:10.2307/j.ctv36xvjfc

[CIT0029] Martin, W. (2012). Visualizing risk: Health, gender and the ageing body. Critical Social Policy, 32(1), 51–68. doi:10.1177/0261018311425980

[CIT0030] McKay, M. H., Pickens, N. D., Medley, A., Cooper, D., & Evetts, C. L. (2021). Comparing occupational adaptation-based and traditional training programs for dementia care teams: An embedded mixed-methods study. Gerontologist, 61(4), 582–594. doi:10.1093/geront/gnaa160 33075131

[CIT0031] Meeks, S., Shryock, S. K., & Vandenbroucke, R. J. (2018). Theatre involvement and well-being, age differences, and lessons from long-time subscribers. Gerontologist, 58(2), 278–289. doi:10.1093/geront/gnx029 28398577

[CIT0032] Nikitin, J., & Freund, A. M. (2019). Adaptation process of aging. In R. Fernandez-Ballesteros, J. M. Robine, & A. Benetos (Eds.), Cambridge handbook of successful aging (pp. 281–298). Cambridge University Press. doi:10.1017/9781316677018.018

[CIT0033] Nobis, F. (2015). ‘Dropping a part’: The changing relationship of midlife actors with their profession. About Performance, 13, 197–210.

[CIT0034] Pauluth-Penner, T. (2016). Reminiscence theatre arts as a quality of life initiative for older adults in residential care. Gerontologist, 56(3), 522. doi:10.1093/geront/gnw162.2113

[CIT0035] Pfaller, L., & Schweda, M. (2019). Excluded from the good life? An ethical approach to conceptions of active ageing. Social Inclusion, 7(3), 44–53. doi:10.17645/si.v7i3.1918

[CIT0036] Purvis, J. (2004). Grrrls and women together in the third wave: Embracing the challenges of intergenerational feminism(s). NWSA Journal, 16(3), 93–123. doi:10.2979/nws.2004.16.3.93

[CIT0037] Raisborough, J., Watkins, S., Connor, R., & Pitimson, N. (2021). Reduced to curtain twitchers? Age, ageism and the careers of four women actors. Journal of Women and Aging, 34(2), 246–257. doi:10.1080/08952841.2021.1910464 33835890

[CIT0038] Richards, N., Warren, L., & Gott, M. (2012). The challenge of creating ‘alternative’ images of ageing: Lessons from a project with older women. Journal of Aging Studies, 26, 65–78. doi:10.1016/j.jaging.2011.08.001

[CIT0039] Rubenstein, R. (2001). Feminism, eros, and the coming of age. Frontiers, 22(2), 1–19. doi:10.2307/3347050

[CIT0040] Saltmarsh, E. (2018). My Turn Now. Representage. [Performance viewed November 19, 2018, The Union Theatre, Southwark, London].

[CIT0041] Sanders, J. (2006; 2016) Adaptation and appropriation. Routledge. doi:10.4324/9780203087633

[CIT0042] Segal, L. (2013). Out of time: The pleasures and perils of ageing. Verso.

[CIT0043] Woodward, K. (1999). Inventing generational models: Psychoanalysis, feminism, literature. In K. Woodward (Ed.), Figuring age: Women, bodies, generations (pp. 149–170). Indiana University Press.

